# Bombay Blood Group Phenotype Misdiagnosed As O Phenotype: A Case Report

**DOI:** 10.7759/cureus.45555

**Published:** 2023-09-19

**Authors:** Hira Qadir, Muhammad Omar Larik, Muhammad Ashhal Iftekhar

**Affiliations:** 1 Department of Pathology, Henry Ford Health System, Detroit, USA; 2 Department of Medicine, Dow International Medical College, Karachi, PAK

**Keywords:** rare blood group, abo blood groups, blood group, hemolytic transfusion reaction, bombay blood group

## Abstract

Bombay blood group is a rare type that was initially identified in the city of Bombay, India. It is characterized by the presence of serum antibodies anti-A, anti-B, and anti-H, which can cause agglutination in all blood groups within the ABO system. The clinical importance of the Bombay blood group lies in its inability to receive transfusions from other blood groups. In this case report, we present a case of a young male who was initially misdiagnosed as having an O phenotype, resulting in a hemolytic transfusion reaction. This case highlights the diagnostic and therapeutic challenges associated with rare blood phenotypes.

## Introduction

Blood group antigens have a vitally important role within the medical field, especially with respect to blood transfusion. The most well-known and clinically relevant classification system includes the ABO groups. In 1952, an unusual finding was first reported in the city of Bombay, India, with the presence of serum anti-A, anti-B, and anti-H antibodies showing the capability of agglutinating all the blood groups within the ABO system [1]. Thus, this resulted in the discovery of the Bombay blood type. This is considered to be a significantly rare blood type of the ABO system, with prevalence estimated to be 1 in 1,000,000 in European regions, or up to 1 in 10,000 in Indian regions, with heightened prevalence in the Southern and Western regions of India [1,2].

The Bombay phenotype arises from a point mutation in the H gene, resulting in the improper production of the functional protein needed for H antigen synthesis, called fructosyltransferase [1,3]. Contrastingly, in patients with the Bombay blood group, there is an intrinsic inability to produce the H antigen due to the protein deficiency. Thus, transfusion of A, B, or O blood types into patients with the Bombay blood group can result in a classic manifestation of acute hemolytic reactions due to the production of anti-H antigen antibodies [4]. Therefore, patients of the Bombay blood group may only receive transfusions of blood or blood components from donors of the same blood phenotype.

The clinical significance of the Bombay blood group includes the inability to transfuse with other blood groups, such as A, B, AB, and especially the O type [2]. This can lead to fatal hemolytic transfusion reactions, with a classic triad of symptoms including fever, flank pain, and red-brown urine, along with the development of extravascular hemolysis [3]. Differentiating the O blood group from the Bombay blood group in a blood bank depends initially on reverse grouping. Bombay individuals show agglutination with O cells due to the presence of anti-H, in contrast to the O blood group [4]. Therefore, reverse grouping is extremely important to establish the correct ABO type and to resolve any ABO blood discrepancy. In this case report, the patient was initially misdiagnosed as an O blood group, subsequently receiving a transfusion with a non-Bombay blood group after surgery, ultimately leading to the development of a hemolytic transfusion reaction.

## Case presentation

A 32-year-old male with no known underlying medical conditions was involved in a road traffic accident, where he was admitted to another institute for an exploratory laparotomy and mesenteric tear repair. Postoperatively, he received packed red blood cell transfusions. His blood type was determined to be O Rh-positive. A day later, the patient presented to the emergency department of our institute with complaints of severe abdominal pain and decreased urine output. The laboratory parameters on admission to the emergency admission of the patient are illustrated in Table 1, revealing acute kidney injury. The urine analysis results revealed >20 red blood cells, and his peripheral smear revealed spherocytes, polychromasia, and nucleated red blood cells. A blood sample was received in the blood bank for grouping and cross-match. Manual tube testing was used to determine the patient’s ABO type. The forward grouping showed no agglutination with monoclonal anti-A, anti-B sera, and reversed grouping using A, B, and O cells showed a +4 agglutination reaction with A, B, and O cells. Blood grouping results were ultimately indicative of the Bombay blood group, with the subsequent testing with anti-H lectin sera revealing the lack of agglutination. Saliva testing could not be performed due to the commercial unavailability of testing kits in Pakistan. Subsequently, his blood group phenotype was labeled as Bombay, indicating the patient’s exclusive need to receive blood transfusions from other Bombay donors.

**Table 1:** Laboratory parameters of the patient on admission to the emergency department ALT: alanine aminotransferase, AST: aspartate aminotransferase, ALP: alkaline phosphatase, LDH: lactate dehydrogenase

Parameters	Values at admission	Reference ranges
Hemoglobin (g/dL)	6.6	13.0-17.0
Hematocrit (%)	19	40-50
Mean corpuscular volume (fL)	79	80-100
Mean corpuscular hemoglobin (pg)	27	27-31
White blood cell count (x 10^9^/L)	5.0	4.0-10.0
Platelet count (x 10^9^/L)	146	150-400
Reticulocyte count (%)	3.0	0.5-2.5
Total bilirubin (mg/dL)	2.0	0.2-1.2
Direct bilirubin (mg/dL)	0.6	≤0.3
Indirect bilirubin (mg/dL)	1.4	0.2-0.8
ALT (U/L)	23	≤50
AST (U/L)	18	≤50
ALP (U/L)	95	50-300
LDH (U/L)	2043	140-280
Creatinine (mg/dL)	6.0	0.6-1.2
Blood urea nitrogen (mg/dL)	89	6-24

Based on the patient's clinical history, it was concluded that he experienced a hemolytic transfusion reaction due to a mismatched transfusion. Eventually, the patient required a blood transfusion, but unfortunately, the institutional registry did not have any Bombay blood donors present. Therefore, the screening of family members was conducted, and irradiated red blood cells were successfully transfused from his siblings with the same Bombay blood group. His laboratory parameters improved thereafter. He and his family members were then comprehensively counseled about their rare blood type. Due to financial constraints, the patient was discharged against medical advice.

## Discussion

There is a reasonable similarity between the Bombay blood phenotype and the O group. This is due to the absence of the A and B antigens, which mimic the O phenotype; however, it is incredibly important to consider the presence of the H antigen in all of the ABO blood types. Thus, transfusion of A, B, or O blood types into patients with the Bombay blood group can result in a classic manifestation of acute hemolytic reactions due to the production of anti-H antigen antibodies [3]. Therefore, patients of the Bombay blood group may only receive transfusions of blood or blood components from donors of the same blood phenotype. The comparison of forward and reverse grouping between Bombay and the O phenotype is illustrated in Table 2.

**Table 2:** The comparison of forward and reverse grouping between Bombay and O phenotype

Blood phenotype	Forward grouping			Reverse grouping		
	Anti-A	Anti-B	Anti-AB	A cells	B cells	O cells
Bombay type	-	-	-	+	+	-
O type	-	-	-	+	+	+

Additionally, another similar and rare blood type is the para-Bombay blood group, with only a handful of cases being reported. The prevalence of this blood group is unexplored in many regions of the world due to its rarity and the insufficiency of available data. At a molecular level, the para-Bombay blood group is characterized by the lack of ABH expression on RBCs, with some expression of ABH antigens in bodily secretions [4,5]. In contrast, the standard Bombay blood group does not have any ABH antigenic expression on both RBCs and secretions.

There are several therapeutic challenges for patients with the rare Bombay blood group that must be highlighted. The arrangement of donor Bombay blood is extremely difficult, especially in regions where the prevalence and identification of this rare blood group are low or unavailable. In such cases, there have been different strategies explored for such patients, including the use of fresh frozen plasma, crystalloid and colloid infusion, and autologous blood transfusion [6]. Moreover, the role of normovolemic hemodilution as an alternative to transfusion during emergency surgery has also shown effectiveness, particularly in pregnant women [7]. In another case, a Bombay group patient underwent hand surgery and was not able to retrieve Bombay blood from the institute and/or her relatives, highlighting a common issue faced by such patients. However, in light of the stable hemoglobin and progressive recovery, the patient was successfully discharged without the need for transfusion, instigating the possibility of opting out of transfusion in non-emergent settings [8]. These strategies have shown success in the previously mentioned case reports; however, the lack of concrete evidence and established guidelines or protocols raises the need for future research and recommendations on this challenge. The current recommendation encourages the practice of appropriate clinical decision-making, reduction of peri-operative and post-operative blood losses, and engagement in nationwide planning and action [8].

Ultimately, it is imperative to investigate the patient’s past medical and family history before performing any invasive procedures. Patients presenting with any history of previous, eventful post-transfusion reactions or a positive family history of such acute hemolytic reactions post-transfusion, must be further investigated prior to any surgical procedure to ensure the correct blood typing. Moreover, the creation and maintenance of national rare blood group registries is vital in order to avoid the risk of incompatible transfusions, which may result in further complications in patients who are already critically ill. The role of national registries becomes highly essential in emergency or traumatic situations, where there is insufficient time and resources to perform testing for rare blood group types. It is also recommended to immediately start screening the patient’s family, especially the first-degree relatives, for rare blood group phenotypes, such as the Bombay blood group [6]. This strategy may be useful in any unforeseen future emergencies, in cases where the Bombay blood patient requires a blood transfusion, preventing the consequent risk of an incompatible transfusion.

## Conclusions

In summary, the Bombay blood group phenotype may clinically resemble the O phenotype, due to the lack of A and B antigens in the forward grouping. Individuals with the Bombay blood group can only receive blood transfusions from Bombay donors due to the presence of powerful anti-H antibodies in their serum. This case emphasizes the significance of accurate diagnosis of this rare blood type and also highlights the importance of family screening, counseling, and the establishment of a local Bombay blood donor registry for therapeutic management.
